# Oscillating latent dynamics in robot systems during walking and reaching

**DOI:** 10.1038/s41598-024-61610-5

**Published:** 2024-05-19

**Authors:** Oiwi Parker Jones, Alexander L. Mitchell, Jun Yamada, Wolfgang Merkt, Mathieu Geisert, Ioannis Havoutis, Ingmar Posner

**Affiliations:** 1 Applied AI Lab, Oxford Robotics Institute, University of Oxford, Oxford, UK; 2 Dynamic Robot Systems Group, Oxford Robotics Institute, University of Oxford, Oxford, UK

**Keywords:** Engineering, Computer science

## Abstract

Sensorimotor control of complex, dynamic systems such as humanoids or quadrupedal robots is notoriously difficult. While artificial systems traditionally employ hierarchical optimisation approaches or black-box policies, recent results in systems neuroscience suggest that complex behaviours such as locomotion and reaching are correlated with limit cycles in the primate motor cortex. A recent result suggests that, when applied to a learned latent space, oscillating patterns of activation can be used to control locomotion in a physical robot. While reminiscent of limit cycles observed in primate motor cortex, these dynamics are unsurprising given the cyclic nature of the robot’s behaviour (walking). In this preliminary investigation, we consider how a similar approach extends to a less obviously cyclic behaviour (reaching). This has been explored in prior work using computational simulations. But simulations necessarily make simplifying assumptions that do not necessarily correspond to reality, so do not trivially transfer to real robot platforms. Our primary contribution is to demonstrate that we can infer and control real robot states in a learnt representation using oscillatory dynamics during reaching tasks. We further show that the learned latent representation encodes interpretable movements in the robot’s workspace. Compared to robot locomotion, the dynamics that we observe for reaching are not fully cyclic, as they do not begin and end at the same position of latent space. However, they do begin to trace out the shape of a cycle, and, by construction, they are driven by the same underlying oscillatory mechanics.

## Introduction

Biology has served as inspiration for artificial intelligence and robotics from the founding of these fields. Yet many engineering solutions for complex planning and control tasks make use of non-biologically plausible optimisation schemes and are customised for specific robot platforms. One reason for this divergence is that we do not fully understand, and therefore are not able to directly replicate, how brains solve complex planning and control problems in tasks such as manipulation and locomotion. Of course this does not mean that neuroscience has nothing to offer, especially as the frontiers of neuroscience are continuously expanding.

**Figure 1:**
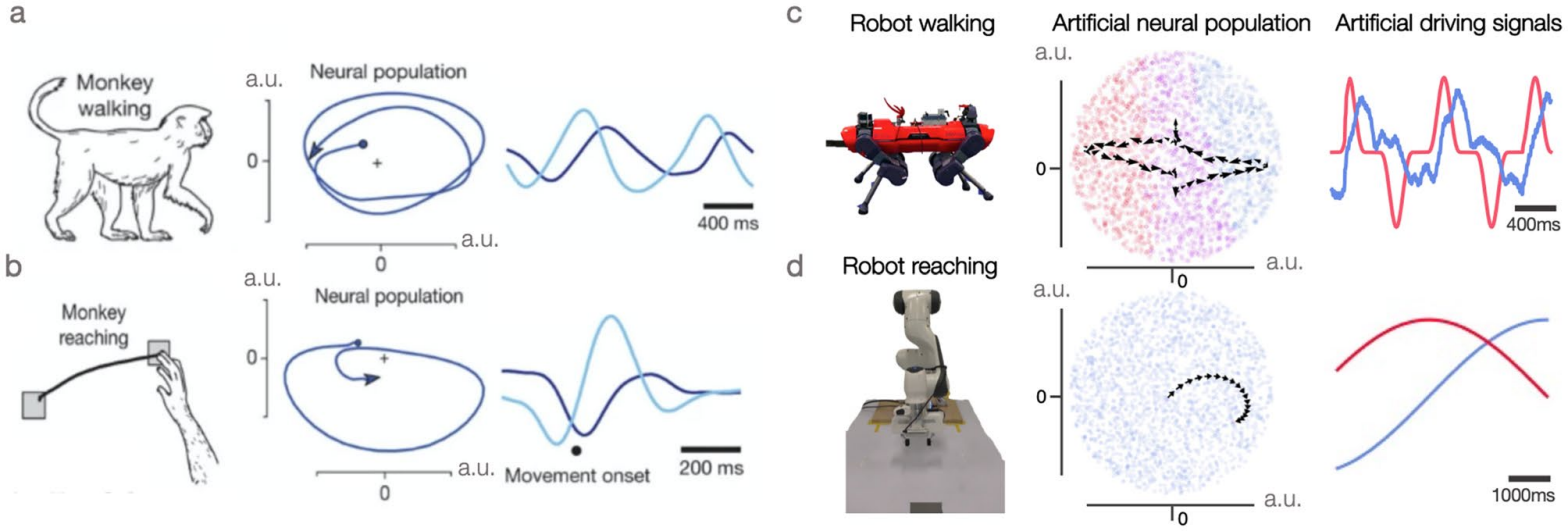
Rotational trajectories in monkeys and robots while walking and reaching. In the process of learning a structured latent-space for robot locomotion^1^, we discover oscillatory dynamics that bear an intriguing resemblance to those observed in the monkey motor cortex^2^. We show for the first time that these dynamics can be used to drive a robot arm during the non-periodic task of reaching. (**a**) Monkey walking. (**b**) Monkey reaching. (**c**) Robot walking. (**d**) Robot reaching. Note the broad analogy between corresponding monkey and robot panels. Each contains an image of the behaviour (walking or reaching), the associated rotational trajectories, and equivalent neural oscillations. Although reaching is a non-periodic behaviour, rotational trajectories are shown for both neural populations in the monkey motor cortex (axes represent principal components) and artificial neurons in the robot model (axes represent latent dimensions). Further details are explained in the text. Monkey panels adapted with permission from prior work^2^.

The present work takes inspiration from two recent developments, one in systems neuroscience^2^ and another in robotics^3^. An analogy between them is teased in Fig. 1. On the robotics side, Mitchell et al.^3^ learned two things by using a variational autoencoder (VAE)^4,5^ to encode the states (e.g. joint positions) of a legged robot. First, they found that different leg configurations correspond to different positions in the learned latent space. The second discovery was that the different latent states (which represent different foot configurations) are organised so that walking corresponds (roughly) to elliptical trajectories in a low-dimensional (2D) projection of the latent space. The existence of elliptical trajectories did not need to be the case: walking might have corresponded to more erratic wanderings through latent space.

The authors next showed that synthetic oscillations can be used to generate robot behaviour^1^. In an investigation of latent dynamics in the trained VAE during walking, one dimension of latent space stood out: it oscillated regularly and in synchrony with the robot’s walking. The authors showed that by overwriting this latent dimension with a synthetic drive signal (imagine a parameterised sine wave), they could continuously control the robot’s behaviour. For example, changing the amplitude of the sine wave corresponded to how high the robot raised its legs^1,6^. The shape of the sine wave thus had interpretable effects on robot behaviour.

On the neuroscience side, Churchland et al.^2^ report similar elliptical trajectories in neural activity recorded from the motor cortex of monkeys while performing tasks like walking and reaching^2^. These elliptical cycles were observed in a low-dimensional projection (2D) of the population neural code. Cyclic patterns in the brain are less surprising for walking than for reaching, as walking is a cyclic behaviour. But reaching is not obviously cyclic. Therefore, we ask whether similar oscillations drive reaching behaviour in a robot. Motivated by the example from robot locomotion^1,3,6^, our aim in this study is to see if we can train a VAE for a robot arm and then control it with oscillating drive signals (see Fig. 1).

Our primary contribution is to demonstrate that the state of the robot arm can be both *inferred* and *controlled* in the latent space, using synthetic drive signals which are rotational by construction, being out of phase by $\pi /2$ radians (Fig. 1d). Consequently, the general approach used for legged robots^1,3,6^ (Fig. 1c) can be extended to reaching. When plotted in 2D, the drive signals for both reaching and walking in robots bear an intriguing resemblance to those observed in living brains^2^ (Fig. 1b,a). We are inspired by prior work^7,8^ which finds that rotational dynamics in the hidden layers of recurrent neural-networks (RNNs)^9,10^ can solve reaching tasks *in-simulation*. In contrast to prior works, we choose an alternative architecture to an RNN and apply rotational dynamics in a learnt representation to solve control tasks on a *real* robot platform, the Frank Emika Panda arm^11^. As a result, our research focuses on the real-world control for both locomotion and manipulation. Indeed, simulations make simplifying assumptions. For example, physical implementations expose systems to variables like sensor noise, mechanical wear, and unmodelled environmental interactions. Moreover, demonstrating that an idea works on a physical platform is important in fields like robotics where the end-goal often includes deployment in real-world situations. In addition, we show that oscillatory movements in separate dimensions of the manipulator’s latent space control movements both longitudinal (forward/backward) and lateral (left/right) movements independently. This mirrors discoveries in the robot locomotion domain where key gait characteristics such as footswing lengths and heights are disentangled within the representation^3,6^.

Our secondary contributions are minor: we suggest a few ways of investigating the learned representations in our models. It would be easy to exaggerate the importance of these investigations by making too much of the analogy between rotational dynamics in biological and artificial neural networks. Shared computational principles may underlie both systems. But we do not yet understand these principles, so it is too soon to draw strong conclusions (e.g. infer things about brains based on interpretations of robot systems). We therefore present the present work as preliminary.

The remainder of the paper is organised as follows. "Materials and methods" describes the methods for training VAEs on legged robots and robot arms. It describes the methods for inferring latent states with rotational dynamics and for injecting synthetic drive signals into the latent states to control robot behaviour. "Results" presents the results. We see that training latent space models for robot locomotion and manipulation tasks results in intriguing similarities to biological systems. Nonetheless, our primary contribution is to demonstrate that these models can be used for robot control. "Discussion" ends the paper with a discussion of the results in the context of robot control and systems neuroscience.

## Materials and methods

In this section we detail how to utilise VAEs^4,5^ to create latent spaces in which cyclic dynamics emerge for the locomotion and reaching. Our method draws together previous work^1,6^ while extending it with a view to highlighting the biological inspiration of a latent-space approach to robot control. We first present common foundations for both the locomotion and reaching systems ("A general framework for locomotion and reaching") before presenting the locomotion-specific architecture ("Locomotion-specific architecture") and manipulator-specific architecture ("Manipulator-specific architecture"). Next we describe the construction of drive signals for locomotion ("Drive signals for locomotion"), followed by drive signals for manipulation ("Drive signals for manipulation"). Finally, we detail the data collection for locomotion ("Data collection for locomotion") and manipulation ("Data collection for manipulation"), followed by a short note on close-loop control ("Closed-loop control"). For this study, we used the highly-dynamic ANYmal quadruped, both B and C variants, for the walking system (see Fig. 1c). For reaching, we employed a Franka Panda arm (Fig. 1d). The Franka Panda arm is a 7 degree of freedom (DOF) manipulator.

### A general framework for locomotion and reaching

As input to the VAE models^4,5^, we use a concatenation of measurable robot states sampled in time. These are quantities that are either required for inference or for variables we wish to control. For example, we encode the quadruped’s end-effector contact forces in order to infer the contact state, as well as the joint configurations as these are decoded and sent to the tracking controller. *End-effectors* are contact points with the environment such as grippers for robot arms, or the points at which legs contact the ground. A *join configuration* in robotics refers to the specific angles (e.g. in degrees or radians) of all the joints in a robotic arm or leg at a given moment.

Let the input to the VAE ${\textbf{X}}_k$ be a history of the previous *N* robot states ${\textbf{x}}_k$ stacked on top of each other. A history of previous states is used as it is necessary to encode how the motion of the robot changes over time to infer the momentum of the robot at the current time step *k*. The control frequency $f_{\textrm{c}}$ is the frequency the VAE-planner operates at, providing updated predictions of future robot motion. For the quadruped, this frequency is 400 Hz. For the manipulator, it is 20 Hz. To understand the different control frequencies, note that the quadruped may need to make frequent adjustments to stay upright. By contrast, the manipulator operates in a more controlled environment, so its planner does not need to sample as frequently. The states which make up the encoder’s input are downsampled to a lower encoder-frequency $f_{\textrm{enc}}$ compared to the control frequency. We thus introduce the ratio $r=f_{\textrm{enc}}/f_{\textrm{c}}$ to create the input:

1 $$\begin{aligned} {\textbf{X}}_k = [{\textbf{x}}^\top _{k-r(N-1)}, \ldots , {\textbf{x}}^\top _{k-r}, {\textbf{x}}^\top _k]. \end{aligned}$$

An alternative formulation would be to use the derivative of the robot states instead (e.g. velocity and acceleration). But these are very noisy quantities in practise, and implicitly inferring them helps with the domain transfer from simulation to real robot.

Once the input ${\textbf{X}}_k$ is constructed, we encode both a mean latent-variable $\mathbf {\mu }_k$ and a variance $\mathbf {\Sigma }^2_k$. The mean and variance serve to parameterise the prior distribution, in this case a zero-mean and unit-variance Gaussian. We can sample the latent variable ${\textbf{z}}_k \sim N(\mathbf {\mu }_k, \mathbf {\Sigma }^2_k)$. Together with the input action ${\textbf{a}}_k$, this forms the input to the decoder. This is also the input to the performance predictor $g_{\textrm{pp}}$, which is optional and can be used to enforce semantic constraints^1,12–14^. In the locomotion case a performance predictor is used to predict which of the robot’s feet are in contact with the ground. The robotic arm did not require any constraints in our reaching experiments. The decoder output $\hat{{\textbf{X}}}^{+}_k$ is formed as

2 $$\begin{aligned} \hat{{\textbf{X}}}^{+}_k = [\hat{{\textbf{x}}}^\top _{k}, \hat{{\textbf{x}}}^\top _{k+1}, \ldots , \hat{{\textbf{x}}}^\top _{k+M}]. \end{aligned}$$

The VAE is a generative model. To train it, we maximise the evidence lower bound (ELBO)^4,5^. This is a lower bound on the evidence of the input distribution and is constructed of two terms. One of these is the reconstruction loss between the target values ${\textbf{X}}^{+}_k$ and the predicted values $\hat{{\textbf{X}}}^{+}_k$. The other term is the is the KL-divergence between the predicted distribution, $q({\textbf{z}}_k|{\textbf{X}}_k)$, and the prior distribution, $p({\textbf{z}}_k)$. We use Guassian distributions for the prior, posterior, and likelihood functions. The loss function thus takes the form

3 $$\begin{aligned} \mathcal {L}_{\textrm{ELBO}} = \text {MSE}({\textbf{X}}^{+}_k, \hat{{\textbf{X}}}^{+}_k) + \beta \mathcal {D}_{\textrm{KL}}[q({\textbf{z}}_k|{\textbf{X}}_k) || p({\textbf{z}}_k)], \end{aligned}$$

where the mean-squared error (MSE) is used for the reconstruction loss, and $\beta$ is a hyperparameter that trades off between how well the VAE reconstructs the input data and how much it regularises the latent space to encourage disentanglement of latent factors^15^.

### Locomotion-specific architecture

**Figure 2:**
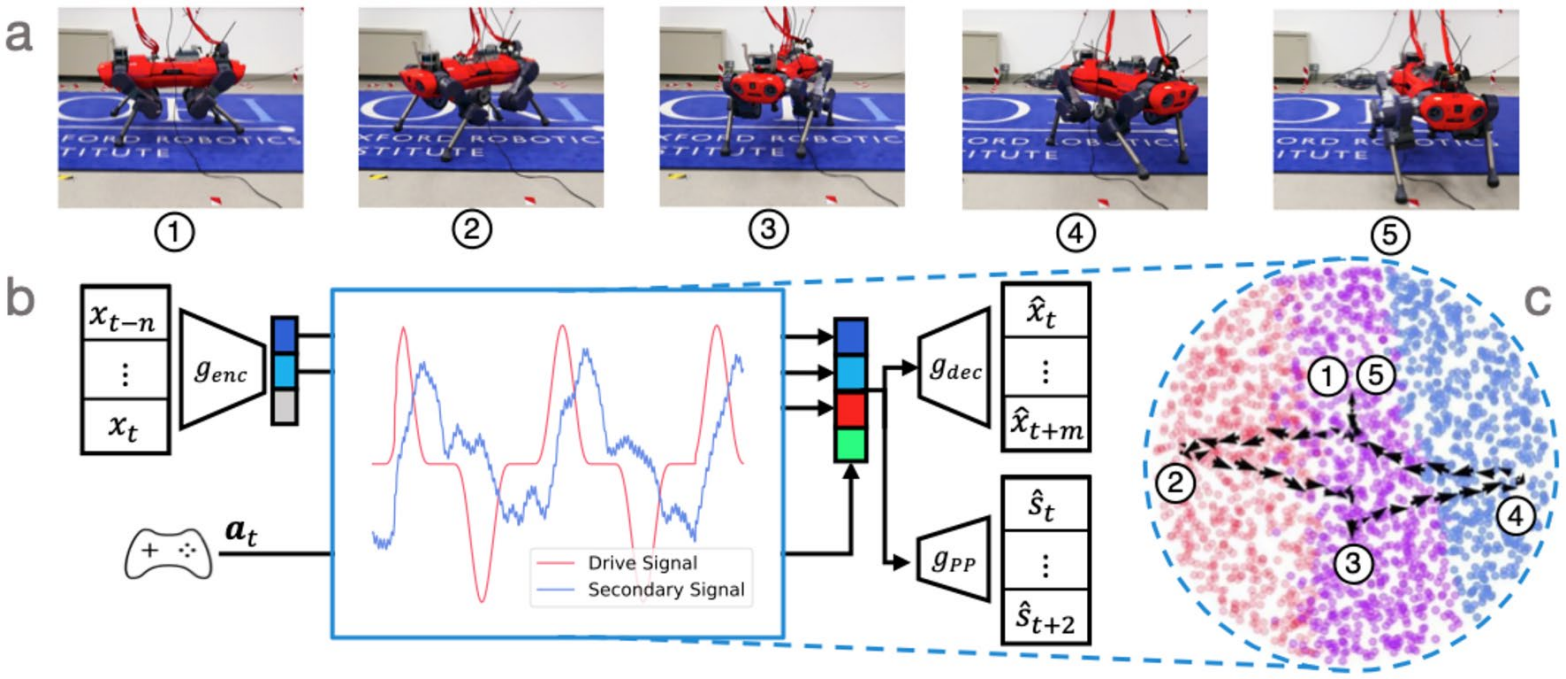
Robot locomotion induced by artificial drive signals. (**a**) Quadruped locomotion. Numbered snapshots correspond to points in the learned latent space. (**b**) Injection of drive signal into learned latent space. Raw sensor data is encoded into latent space prior to the injection of an oscillatory drive-signal (red). During closed-loop operation, we encode the robot states and can infer a secondary signal (blue), which is $\pi /2$ out of phase with the drive signal. Decoding the updated latent variable along with the desired base-twist action ${\textbf{a}}_k$ results in locomotion trajectories which are tracked by a whole-body controller. (**c**) Rotational trajectory in learned latent space. Plotting the drive signal against the secondary signal reveals that locomotion follows a limit cycle in latent space (black arrows). Numbered points in latent space correspond to robot configurations seen in quadruped locomotion, as described earlier in this caption. Colour-coding of points in latent space correspond to foot configurations, to be described in Fig. 7.

A few domain-specific modifications are required to learn a model for robot locomotion. Building on the general framework described above, Fig. 2 provides an overview of our specific approach to the locomotion problem. We will first discuss the entire set of inputs required for locomotion planning before explaining how we structure the latent space using performance predictors.

The input robot-state ${\textbf{x}}_k$ is constructed using the joint angles, end-effector positions in the base frame, joint torques, contact forces, gravity body vector, base velocity, and finally the base pose relative to a control frame. In our case, we choose the control frame to the base pose in the world at time step ${k-N+1}$. Inputs are encoded by the VAE $g_{\textrm{enc}}$ and then decoded by the VAE $g_{\textrm{dec}}$. Note that the drive signal $\alpha _t$ is concatenated to the latent vector before decoding.

To predict which of the feet are in contact ${\textbf{s}}_k$, we employ a performance predictor $g_{\textrm{pp}}$^1,12–14^. This performance predictor $g_{\textrm{pp}}$ is a multi-layer perceptron which takes the latent variable ${\textbf{z}}_k$ as input. Similar to the decoder, we predict the current time-step as well as $J-1$ future steps:

4 $$\begin{aligned} \hat{{\textbf{S}}}_k = [\hat{{\textbf{s}}}^\top _{k}, \hat{{\textbf{s}}}^\top _{k+1}, \ldots , \hat{{\textbf{s}}}^\top _{k+J-1}]. \end{aligned}$$

We train the VAE and the performance predictor $g_{\textrm{pp}}$ in conjunction with each other to encourage structure that is informed by both in the latent space. We used binary cross-entropy (BCE) for the performance predictor loss. The total loss for locomotion is therefore

5 $$\begin{aligned} \mathcal {L} = \mathcal {L}_{\textrm{ELBO}} + \gamma \text {BCE}({\textbf{S}}_{\textrm{k}}, g_{\textrm{pp}}(\hbox {z}_{\textrm{k}})), \end{aligned}$$

where $\gamma$ is a weighting between the $\mathcal {L}_{\textrm{ELBO}}$ loss and the BCE.

**Figure 3:**
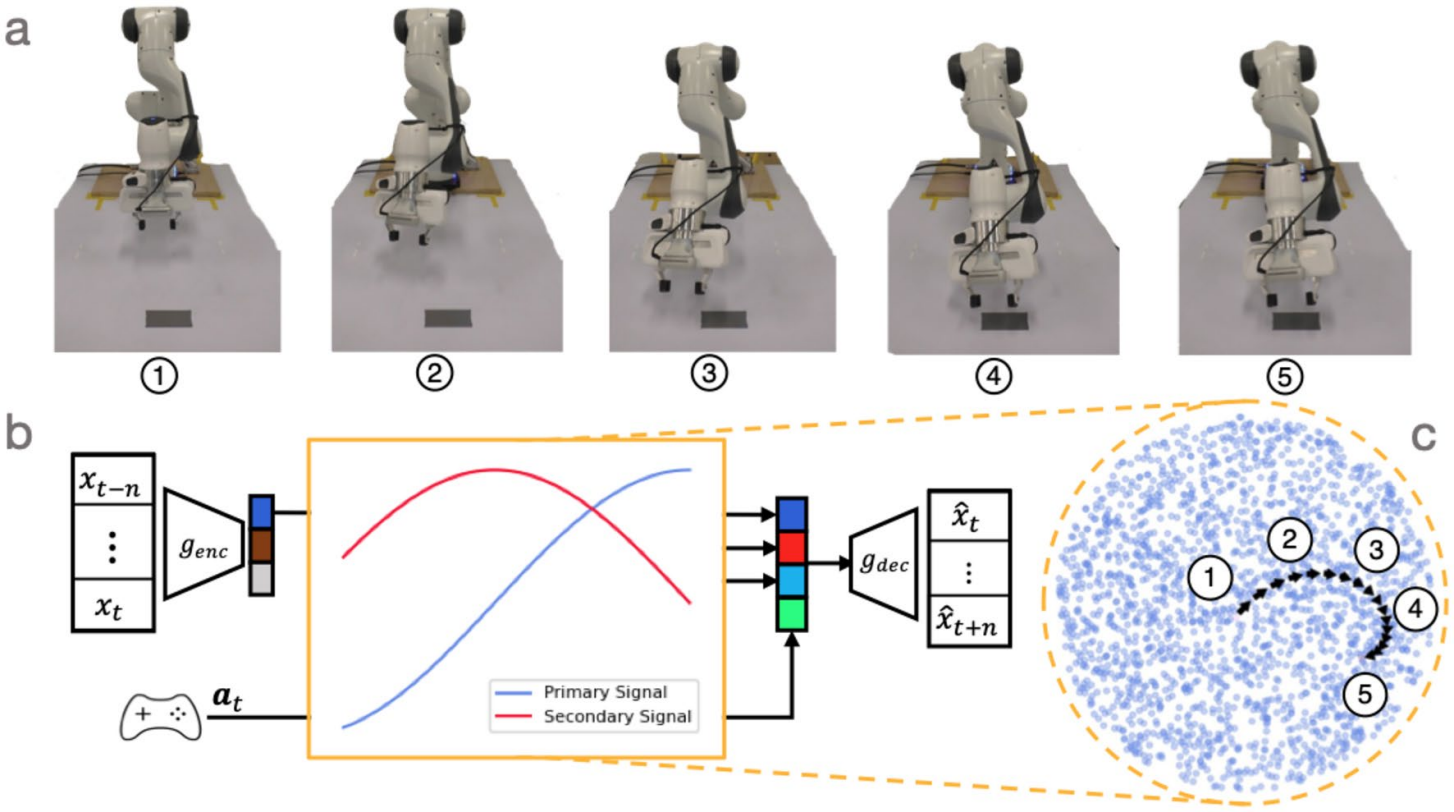
Robotic arm reaching induced by artificial drive signals. In contrast to locomotion, training the VAE for reaching does not require the performance predictor. **a** The outcome of the robotic arm reaching. The arm end-effector moves towards the gray tape on the table. **b** Two artificial drive signals are injected into the latent space trained with the VAE. The drive signals consist of two components where the secondary signal has a phase lag of $\pi /2$ relative to the first. **c** The latent space with the injected drive signals.

### Manipulator-specific architecture

Figure 3 provides an overview of our approach to reaching. Compared to the locomotion problem, training the VAE on reaching data is relatively simple. For instance, we do not require a performance predictor, as we do not have any contact constraints in this domain.

For reaching, the input robot-state ${\textbf{x}}_{k}$ consists of joint angles, the end-effector position, and the goal end-effector position. Again, inputs are encoded by the VAE $g_{\textrm{enc}}$ and latent vectors are decoded by the VAE $g_{\textrm{dec}}$. The synthetic drive signal $\alpha _t$ is concatenated to the latent vector before decoding. To achieve rotations in 2D, the drive signal is composed of two components: a sinusoid and a secondary signal that has a phase lag of $\pi /2$. This creates rotations in 2D (Fig. 3c).

To realise robotic reaching behaviour via the injection of drive signals, which will be formulated in Eq. 9, we need to form a goal robot-state ${\textbf{x}}_{T}$ that requires goal joint angles. Goal joint angles can be obtained by inverse kinematics or manual specification by a user.

### Drive signals for locomotion

To construct a drive signal for locomotion, we employ a cubed sine ($\sin ^3$) drive signal with parameters for amplitude $A_k$ and phase $\phi _k$:

6 $$\begin{aligned} {\textbf{z}}_{k,d_z} = A_k \sin ^3(\phi _k). \end{aligned}$$

The choice of a cubed sine is based on performance. The phase is parameterised using the time period $T_k$ and the stance counter $\epsilon _k$. The time period controls the quadruped’s swing time, while we utilise the stance counter to artificially extend the duration that the robot has four feet in contact with the ground. Therefore, the phase dynamics update as

7 $$\begin{aligned} \phi _{k+1} = {\left\{ \begin{array}{ll} \phi _k &{} \text {if}\ \phi _k \bmod \pi = 0 \ \text {and}\ k_{\epsilon } < \epsilon _k \\ \phi _k + {2\pi }/{T_k} &{} \text {otherwise} \end{array}\right. } \end{aligned}$$

and, contemporaneously, the counter $\epsilon _k$ propagates:

8 $$\begin{aligned} k_{\epsilon } = {\left\{ \begin{array}{ll} k_{\epsilon } + 1 &{} \text {if}\ \phi _k \bmod \pi = 0 \ \text {and}\ k_{\epsilon } < \epsilon _k \\ 0 &{} \text {otherwise.} \end{array}\right. } \end{aligned}$$

Example drive signals and their corresponding behaviours are presented in Fig. 7b. We utilise a sine cubed function since its gradient is zero when the phase is equal to a multiple of $\pi$. Therefore, when the phase is artificially held constant for $\epsilon _k$ time steps during the stance phase, the drive signal remains smooth. A smooth drive signal, in turn, yields smooth robot trajectories which are easier for the whole-body controller to track.

Given this formalisation, we can vary the drive signal’s amplitude $A_k$, and parameterise the phase $\phi _k$ using $T_k$ and $\epsilon _k$. This results in observable correspondences between the drive signal and robot behaviour, which we describe in the results.

### Drive signals for manipulation

Once the manipulation specific latent-space has been created, we inject a drive-signal into the latent space which enables the robot to reach a desired goal position. To solve the reaching problem, we encode the start position $$z_{\textrm{start}}=\begin{bmatrix}z_{k=0, d_{z}=0} \\ z_{k=0, d_{z}=1} \end{bmatrix}$$ and goal position $$z_{\textrm{goal}}=\begin{bmatrix}z_{k=T, d_{z}=0} \\ z_{k=T, d_{z}=1} \end{bmatrix}$$ into latent space using the VAE’s encoder. With these latent variables and given the time horizon *T*, we introduce the following primary and secondary driving signals at timestep *k* for the robotic arm reaching

$$\begin{aligned} R(t) = \begin{bmatrix} z_{k,0} \\ z_{k, 1} \end{bmatrix} =\begin{bmatrix} A\cos (\phi _{t}) + z_{T, 0}\\ A\sin (\phi _{t}) + z_{T, 1} \end{bmatrix}, \end{aligned}$$

where:

9 $$\begin{aligned} A&= ||(z_{\textrm{goal}}-z_{\textrm{start}})|| \left( 1-\frac{k}{T}\right) \nonumber \\ \phi _{k}&= {{\,\textrm{atan2}\,}}(z_{k=0, d_{z}=1} -z_{k=T, d_{z}=1}, z_{k=0, d_{z}=0}-z_{k=T, d_{z}=0}) \left( 1-\frac{k}{T}\right) . \end{aligned}$$

From this, the latent-space trajectory is decoded and the sequence of robot states are sent to a tracking controller. Control is performed in an open-loop for reaching, as the manipulator is statically stable (its base does not move). This is different than in the legged robot.

### Data collection for locomotion

To train the legged robot model, we create a synthetic dataset of the quadruped walking on flat ground in simulation. The dataset contains 30 min of locomotion sampled at 400 Hz. To vary the locomotion, we randomly sampled base-twist actions which were fed through the planning pipeline resulting in trot locomotion. The locomotion planning pipeline consists primarily of two modules, the first being *Dynamic Gaits* (DG)^16^. This solves for the base-pose and end-effector trajectories. The second module is a whole-body controller (WBC)^17^, which finally converts the base-pose and end-effector trajectory to joint positions, velocities, and torques.

DG solves for the base and feet plans utilising a hierarchical framework, which employs a number of kinematic and dynamic constraints. Firstly, the base-pose optimisation utilises a centroidal dynamics model^18^, and is constrained utilising a zero moment point (ZMP) model^19^. The WBC, which we also utilise to track the VAE trajectories consists of another hierarchical optimisation. For completeness, the constraints enforced are: base-pose tracking, contact creation, friction constraints, and torque limits.

For the simulations, we utilise the *Raisim*^20^ simulator and an actuator network^21^ in order to increase the fidelity of the trajectories. The actuator network uses a learned network to approximate the robot’s series elastic actuators (SEA)^22^. After training, which is performed solely using synthetic data from a single quadruped model, we deploy the same VAE models on two different quadruped robots (ANYmal B and C) in real-world hardware experiments.

### Data collection for manipulation

To train the reaching model, we collect 2000 reaching trajectories from the Robosuite simulator^23^. The resulting trajectories are the output of an expert policy trained with a soft actor-critic^24^. The input to the VAE consists of joint angles, end-effector position, and the goal position in Cartesian coordinates. After training the VAE on the simulated datasets, we deploy the VAE on a real physical Franka Panda arm robot.

### Closed-loop control

For completeness, let us briefly note the use of open and closed-loop controllers. *Open-loop controllers* operate without feedback. Their actions are pre-programmed and not adjusted based on sensor data. *Closed-loop controllers*, on the other hand, require the system to perform constant monitoring and feedback, to adjust based on interactions with the environment.

For closed-loop control, the trained VAEs are used as planners and deployed on the quadrupeds and on the manipulator. This has a number of benefits, such as mitigating external disturbances and counteracting unmodelled dynamics. For example, the ANYmal actuators are SEAs^22^, which are complex systems themselves and are difficult to model analytically, exhibiting dynamic differences between units.

To plan in latent space, we first estimate the current latent variable. Following Eq. 1, we create the encoder input ${\textbf{X}}_k$. We then pass ${\textbf{X}}_k$ through the VAE’s encoder to predict ${\textbf{z}}_k$. The predicted value ${\textbf{z}}_k$ is concatenated with ${\textbf{a}}_k$, which is the desired base-pose twist. Next, we inject our drive signals into latent space, as described above, for the quadruped ("Drive signals for locomotion") and manipulator ("Drive signals for manipulation"). This produces the predicted trajectory snippet, which we decode and send to our tracking controller. For locomotion, the controller is the WBC described in "Data collection for locomotion". The manipulation trajectories are instead tracked using a joint-space position controller. This entire process is repeated at a frequency of 400 Hz on the quadruped robot, and at 20 Hz for the manipulator.

## Results

**Figure 4:**
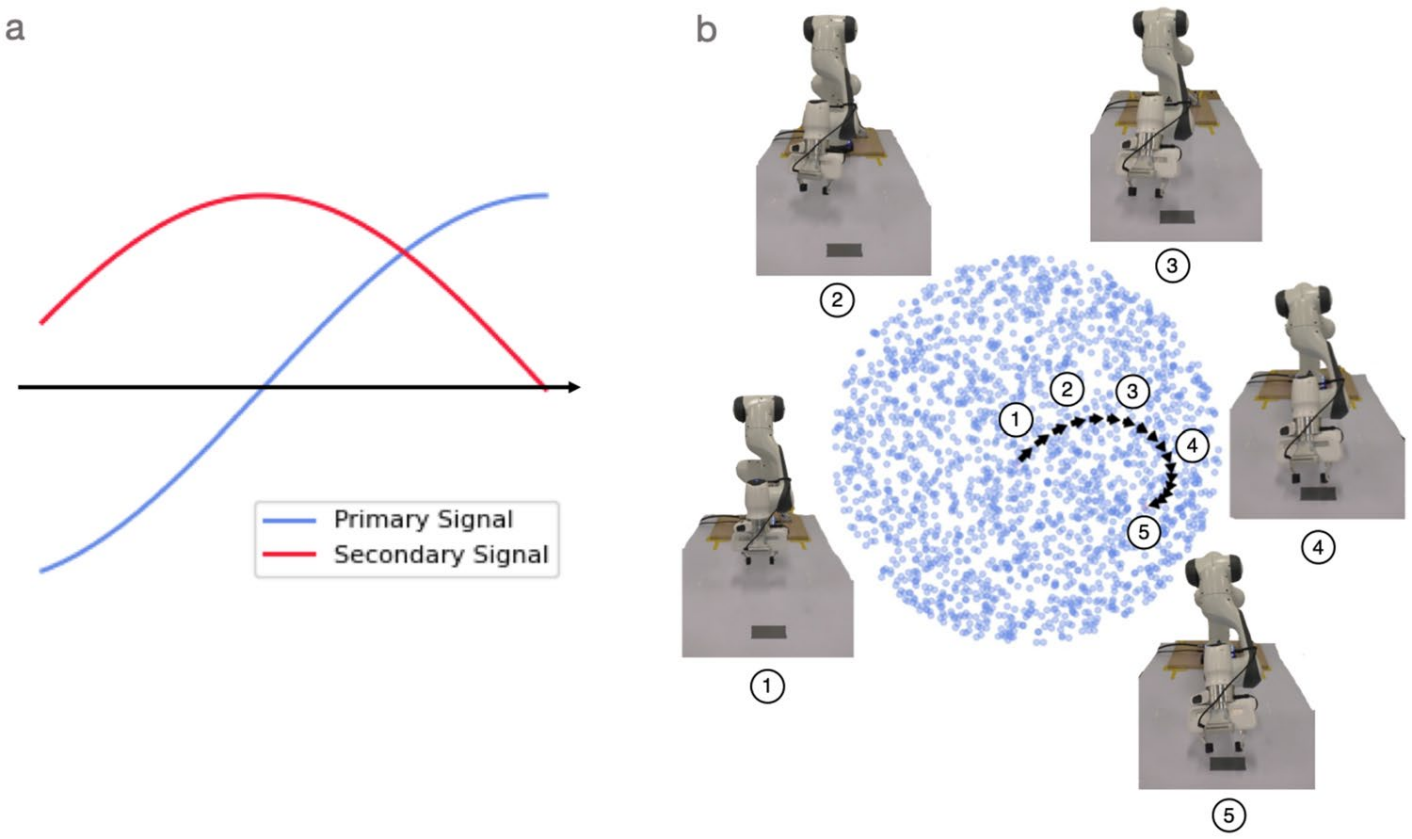
Oscillatory drive signals in the learnt latent-space decode to non-periodic reaching trajectories. (**a**) The two oscillatory drive signals. Both are sinusoidal, with the secondary drive signal being $\pi /2$ out of phase with the primary drive signal. (**b**) Plotting the drive signals against each other reveals a rotational trajectory. The circled numbers along the latent trajectory (black arrows) correspond to the robot arm configurations, which together depict a smooth trajectory from an initial end-effector position to one where it has reached the target (grey square on the table).

Our main result is a proof of concept: we find that the oscillating drive signals, which produce circular motion in latent space, can lead to smooth and directed reaching behaviour in a physical robot. This was already glimpsed in the model architecture (Fig. 3) but is highlighted again here in Fig. 4. This Fig. 4a shows the shape of the oscillatory drive signals, a primary sine wave and a secondary sine wave with its phase shifted by $\pi /2$. These oscillators correspond to a partial cycle in the model’s learned latent space (Fig. 4b), which in turn corresponds to a smooth trajectory in physical space from the arm’s initial position to its goal position. Grey tape appears on the table over the goal position.

**Figure 5:**
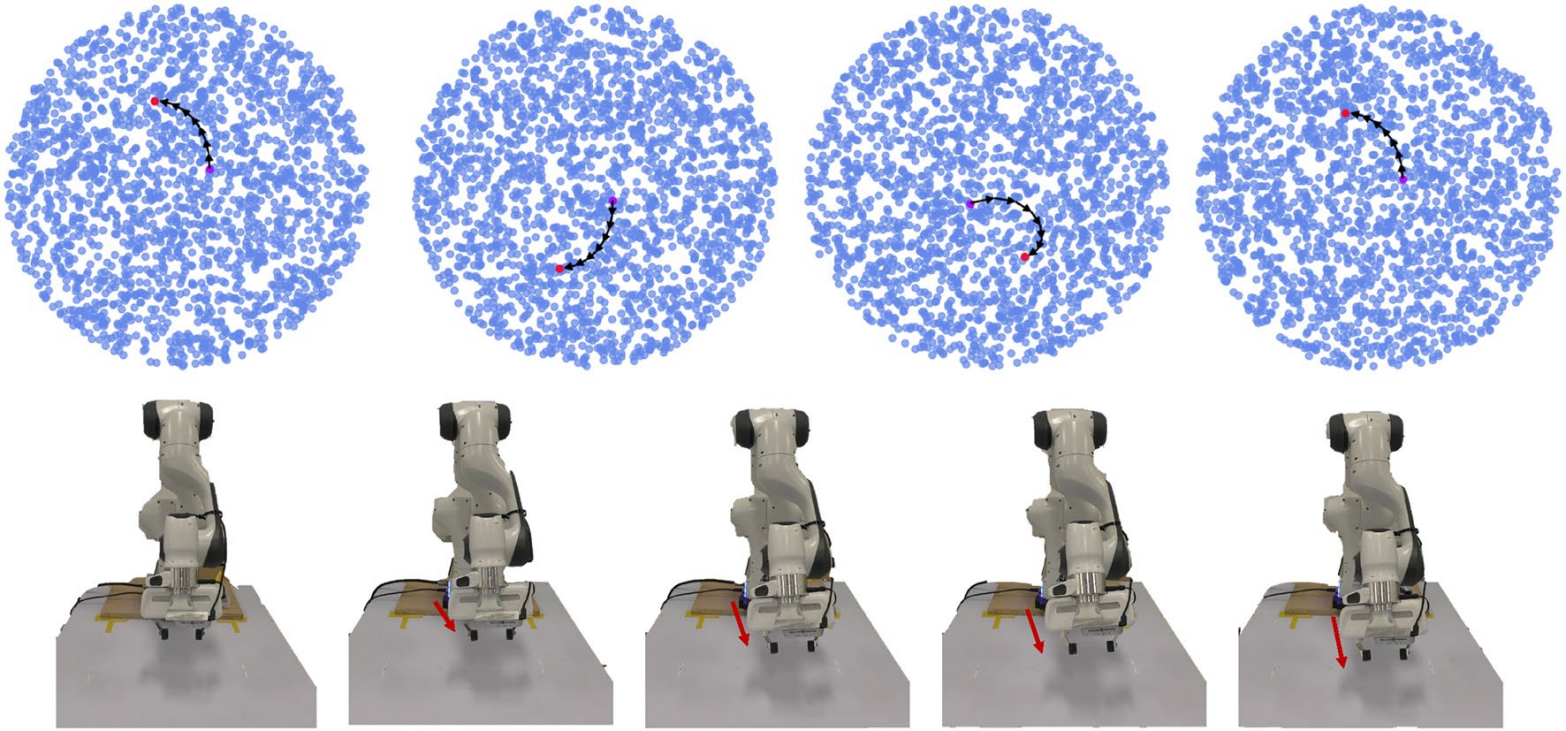
Consistency of latent space structure in robotic arm reaching. A VAE is trained with 4 different seeds. Then its latent space is evaluated by adding drive signals given the same start and goal position for reaching behaviour. All of the latent spaces with the drive signals induces the same successful reaching behavior. This result indicates that the latent space consistently captures the smooth geometry information in the robot workspace, but the latent space represents this workspace differently depending on the seeds.

As a secondary aim, we would like to understand the model’s learned latent representations. To investigate how consistent they are, we trained the reaching model with 4 different random seeds, observing the robot arm trajectories and plotting the latent spaces. These results are presented in Fig. 5. The results reveal that the learned latent spaces differ between random seeds: the latent trajectories do not begin or end in the same places between random seeds. However, each learned latent space reliably enables the arm to reach the same target when controlled with oscillatory drive signals. In effect, the robot arm trajectories were the same across random seeds but we can see variations in the paths traced in latent space.

**Figure 6:**
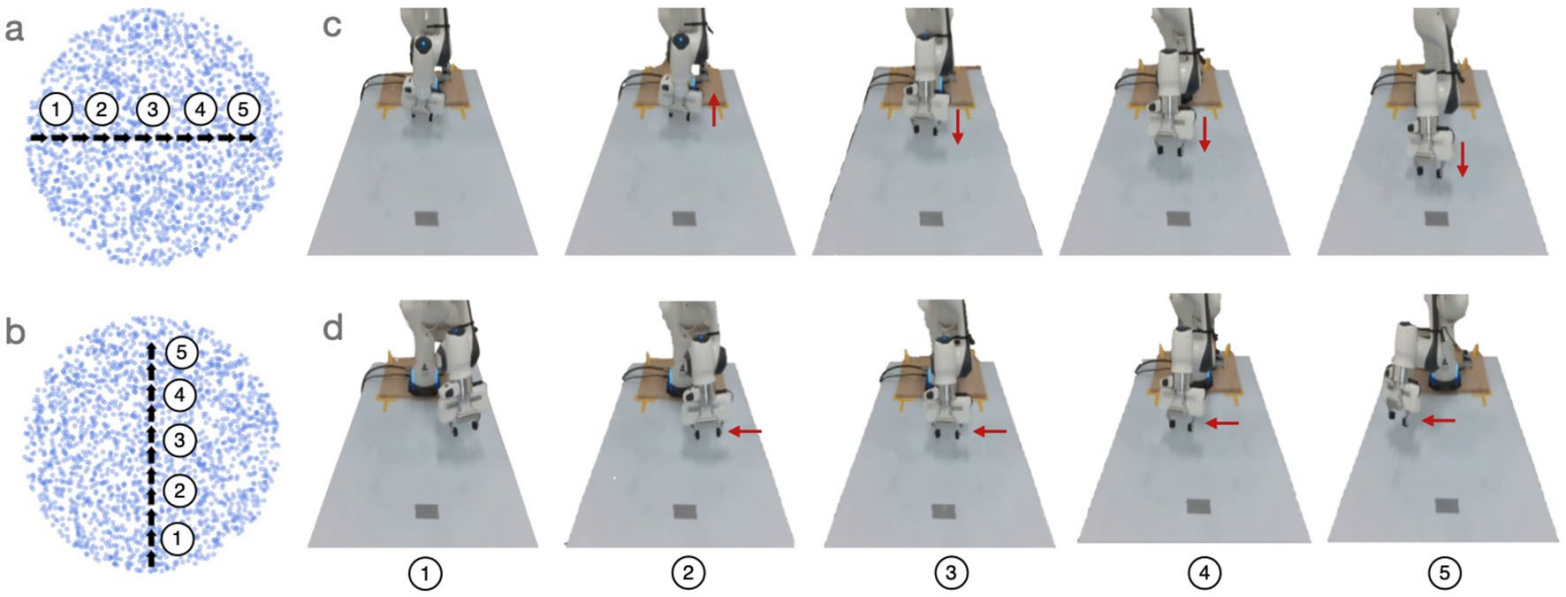
Moving in straight lines across each dimension of the latent space. To explore the learned latent space, we traverse both of its dimensions and decode the corresponding robot arm configurations. (**a**) Traversing the ‘horizontal’ latent dimension. (**b**) Traversing the ‘vertical’ latent dimension. (**c**) Over five snapshots of behaviour for the ‘horizontal’ traversal in latent space, we see the arm in its initial configuration move ‘backward’ before moving ‘forward’ (from the robot’s point of view; see red arrows). (**d**) In the ‘vertical’ traversal of latent space, the robot arm moves more consistently in the same direction (left-to-right from the robot’s point of view; red arrows).

To explore what the dimensions in the learned latent space correspond to, we can sample trajectories in latent space along a line and then visualise the corresponding robot arm configurations. Figure 6a shows five points sampled along the horizontal axis, from left-to-right. Figure 6b shows five points sampled along the vertical axis, from bottom-to-top. The corresponding robot arm configurations are shown in Fig. 6c and d, respectively.

For the horizontal traversal of the latent space, we see the arm initially move ‘backward’ (from the robot’s point of view) before moving ‘forward’ (Fig. 6c). For the vertical traversal, the arm movement is more linear (Fig. 6d). One general observation, from the horizontal traversal, is that the latent dimensions do not necessarily correspond to linear movements in observation space. It would be interesting if this level of analysis generalised from robots to brains, as we argue in the discussion ("Discussion"). However, before continuing to the discussion, let us briefly consider an analysis of the robot locomotion system.

**Figure 7:**
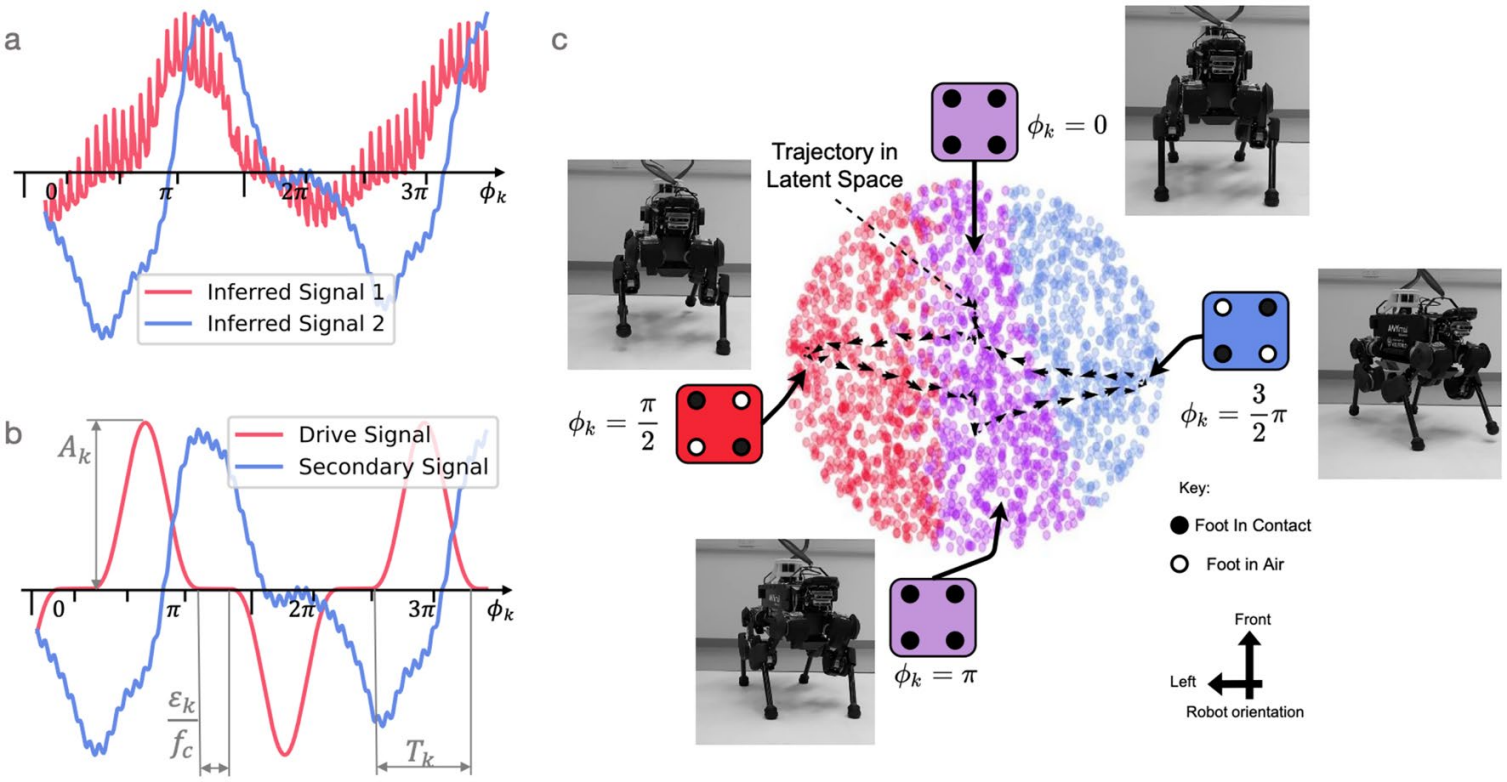
Latent space oscillations, rotation trajectories, and corresponding robot configurations. (**a**) Inferred oscillations. These emerge in an unsupervised generative model trained to observe a set of state space quantities (e.g. joint angles) of an ANYmal B quadruped as it moves. (**b**) Synthetic drive signal. To test our understanding of the primary inferred signal, we replace it with a single sinusoid. This can be shaped by varying a set of parameters at each time step *k*: amplitude $A_k$, phase $\phi _k$, time-period $T_k$, and full-support duration $\epsilon _k/f_c$. We use a cubed sine wave for locomotion to better model the full-support duration which is the period in which all four feet contact the ground. **c** Rotational trajectories and corresponding robot configurations. Plotting the synthetic drive signal against the inferred secondary signal results in rotational trajectories in latent space (black arrows). To aid interpretation, each point in latent space can be decoded to a robot configuration, which we colour code according to one of three cases: all four feet are on the ground (purple); the Left Front and Right Hind feet are on the ground (red); or Right Front and Left Hind feet are on the ground (blue). Points were randomly generated in the latent space and then propagated through the decoder to assign a colour (corresponding to a foot configuration). See text for further details. Figure adapted from prior work^6^.

Prior work shows that similar oscillations can drive robot locomotion^1,3,6^. As presented in the introduction, Mitchell et al.^3^ originally trained a VAE on the states of a walking robot and noticed periodic signals in the inferred latent space. We visualise that here in Fig. 7a. Replacing the primary periodic signal which emerged naturally with a synthetic drive signal that was hand crafted (cf. Fig. 7a,b), the authors found they could control the behaviour of the legged robot. In this case, the secondary signal was inferred by the system (cf. Fig. 3). Plotting the drive signal and inferred secondary signal in 2D, the resulting trajectory in latent space could be related to different robot states (Fig. 7c). Perhaps the deepest understanding of this relationship can be seen from the colour-coding of the latent space, to indicate which regions of latent space correspond to which robot configurations. For example, in 7c, the latent states that are coloured purple correspond to robot configurations in which all four of its feet are on the ground. As the trajectory in latent space cycles in and out of these purple regions, the robot transitions from having all four feet on the ground, to configurations in which different feet are not touching the ground.

**Figure 8:**
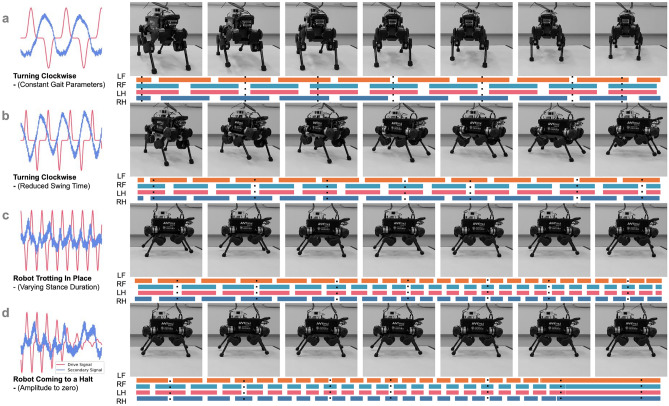
Modulating the amplitude and frequency of the drive signal leads to continuous and interpretable variation in robot gaits. Feet are abbreviated in the contact schedule: LF (Left Front), RF (Right Front), LH (Left Hind), and RH (Right Hind). **a** Robot turning clockwise. **b** Faster clockwise turning with a higher frequency drive signal. **c** Change in drive signal and swing duration over time. **d** Reducing the amplitude of the primary drive signal over time. This ultimately results in the robot halting. Figure adapted from^1^.

Finally, it is possible to control the robot’s behaviour by modulating the drive signal. This can be seen in Fig. 8. For example, Fig. 8d shows what happens when the amplitude of the drive signal is reduced over time. Note that the amplitude of the drive signal controls the step height of the robot. The height of the robot’s steps thus gets smaller as the amplitude of the drive signal gets smaller. Ultimately the steps are too small to overcome friction and the robot halts.

## Discussion

In this paper, we demonstrate that a real-robot manipulator can be controlled using oscillatory signals in a structured latent-representation. We also observe that the properties of these signals (namely the $\pi /2$ phase lag) appear to resemble those found in the brain’s of primates during reaching (see Fig. 1). In learning a latent representation for the control of real robot systems, we find that we can solve robotics tasks using *explicit* rotations (i.e. $\pi /2$ oscillatory drive signals). Prior art has argued for the use of rotational signals in recurrent neural networks^8^, where the internal state (hidden activations) evolves over time and rotations emerge from (or are *implicit* in) the choice of network architecture. We further find structure in the learned latent spaces of our models. For example, inspection of the manipulation latent representation reveals that when oscillations are injected into each dimension of a 2D slice of latent space the robot end effector moves in interpretable patterns, either up and down or side to side (Fig. 6). The consistent emergence of this learned latent space for reaching is verified by training multiple VAEs with four different random seeds and observing the resulting latent structures Fig. 5). This result is similar to the locomotion case where individual latent dimensions encode swing lengths and heights independently as found in prior work^1,6^. Together, these results illustrate the kinds of interpretable structures that emerge in such models’ learned latent spaces.

To compare the legged robot, we followed prior work^1,3,6^ in colour-coding the latent space based on the robot’s foot configurations (Fig. 7c). We also varied the shape of the drive signal and observed how this affects the robot’s behaviour (Fig. 8). Additional exploratory analyses are included in the Supplementary Materials, where we observe the latent space when the legged robot is pushed while walking, deviating from its expected path (Supplementary Figure S1). We also explore how behaviour in the legged robot responds to virtual lesions (Supplementary Figure S2).

In this paper, we extend the VAE-based approach of Mitchell et al.^3^ from walking to reaching. While this approach shares foundational assumptions with theoretical neuroscience, it diverges as well in many details. The shared assumptions are very general: for example, current theories of neuroscience view the brain as a generative model that optimises an objective (free energy) which is equivalent to optimising the ELBO for VAEs^25,26^. In the details, however, stronger links have been forged by work in computational neuroscience^7,8^. Our contribution to this line of work is to extend it from computational simulations to real-world robot platforms: a proof of concept that rotating dynamics in latent space work to drive walking and reaching in robots. The representational learning approach allows one to investigate the latent spaces learned by instances of reaching and walking robots. An important limitations is that we do not claim the learned latent spaces for robots are the same ones learned by animal brains. Rather, we suggest that they represent possible solutions for similar behaviours. Our results build on the recent exploration of VAEs for robot control, which includes the work of Mitchell et al.^1,3,6^ as well as prior work on reaching with VAEs^13^. Prior work on reaching^13^ did not use oscillatory drive signals. The present work therefore helps to unify both behaviours, walking and reaching, under a common biologically-inspired framework.

### Supplementary Information

Supplementary Information.

## Data Availability

The datasets used and/or analysed during the current study available from the corresponding authors on reasonable request.
