# Supplementary material for: Oscillating latent dynamics in robot systems during walking and reaching
Source: Sci Rep. 2024 May 19;14:11434. doi: 10.1038/s41598-024-61610-5 (PMC11102915; doi:10.1038/s41598-024-61610-5)
Supplement: Supplementary file 1 — Supplementary Information. [file 41598_2024_61610_MOESM1_ESM.pdf]

Supplementary Materials

Supplementary Text

Figures S1 and S2

S1 Pushing experiments / The robustness of the VAE-planner

As a supplementary analysis, we show that the VAE-planner is able to detect and reject external disturbances. Figure S1 adapts two figures from our previous work (*6*) that illustrate what

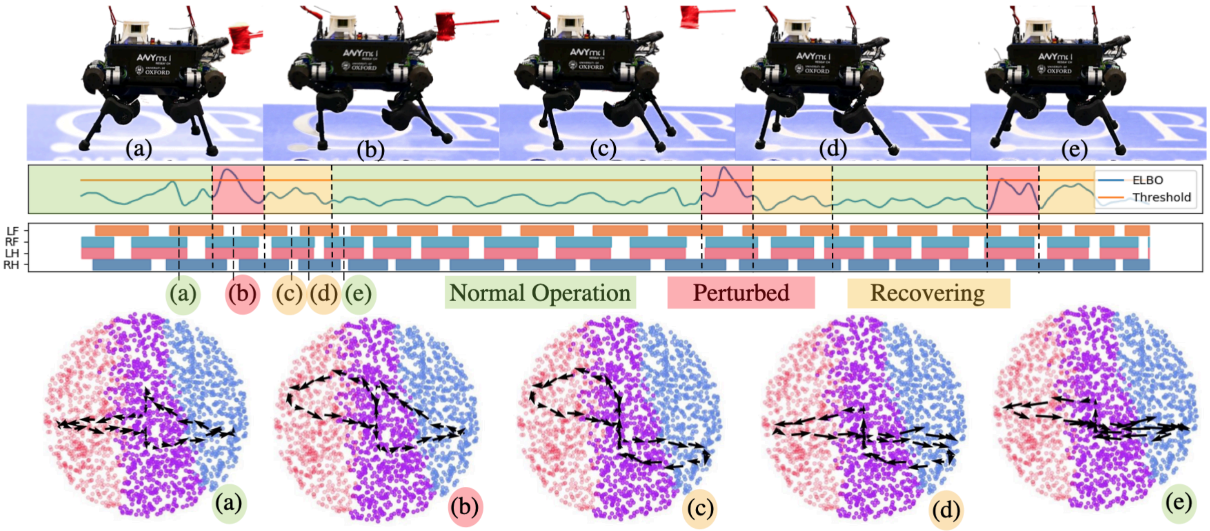

Figure S1: The VAE approach is capable of both detecting and reacting to external disturbances. Here, the external disturbance is a large push from the red broom (top panels). The ELBO corresponding to the encoded input is monitored and if this surpasses a pre-determined threshold, the VAE-planner increases the robot's cadence. Initially, the robot is walking normally and the ELBO is below the threshold (a). At point (b), the robot is pushed. The ELBO rises above the threshold triggering the swing duration to halve. This increase in cadence is captured at point (c) in the contact schedule. After roughly three or four steps, the robot recovers and the swing duration returns to nominal (d) and (e). The bottom row shows the corresponding latent-space trajectories starting with the nominal mode at (a). The external disturbance is clearly visible at point (b) as a large red object (a broom). Adapted from prior work (*6*).

happens to the quadruped's latent space when normal operation is perturbed by an external disturbance (pushing the robot with a broom). Figure S1 shows the robot first detecting the disturbance and then reacting by increasing its cadence to counteract the push. The corresponding latent space plots show how the rotational trajectories are pushed out of orbit but then return towards the original limit cycle. This internal recovery of latent dynamics contrasts with the virtual lesion example, in which the dynamics never recover (Figure S2).

S2 Virtual lesions

Another way to explore the relationship between latent dynamics and behaviour in our VAE approach is to employ virtual lesions. How does progressively damaging the underlying network affect both dynamics and behaviour? To illustrate this, we focus again here on the ANYmal B quadruped robot. By 'lesion' or 'damage', we specifically mean zeroing artificial neurons in the VAE's decoder (illustrated schematically in Figure S2a). In brief, we mask the hidden units in the decoder, randomly setting a subset of them to zero. In the implementation we use cascade failure dropout masks: masks are initialised with ones; mask units are then changed to zero with random probability; finally, zeroed mask units remain zeroed for the remainder of the experiment. The last point means that the virtual lesions are 'progressive', expanding over time without 'recovery'.

Three phases stand out in the exploratory lesion analyses. The first phase is 'behaviour as usual': we inject a drive signal into the latent space and observe the robot walking. In the second phase, we apply progressive lesions to the VAE, using the cascade failure dropout mask approach. Finally, in the third phase we pause lesioning the model and simply observe the latent dynamics and robot behaviour. What happens to the dynamics of the model and behaviour of the robot when the damage stops?

In the latent space, these three phases correspond to distinct dynamics (Figure S2b). In the

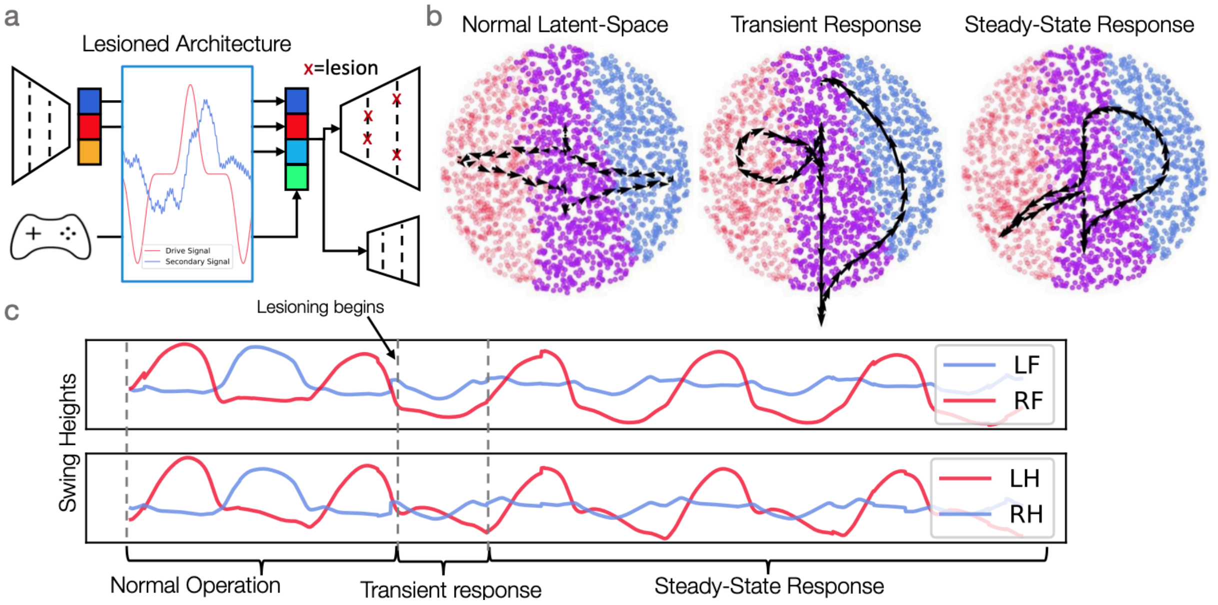

Figure S2: Virtual lesion analysis. (a) We mask hidden units in the VAE's decoder while feeding the drive signal in as usual. Decoder outputs contribute to encoder inputs and thus to the latent space dynamics. Lesions in the decoder are marked with red Xs. (b) Plotting the rotational trajectories in the latent space. We observe three phases in the latent-space dynamics. The first corresponds to normal operation. The second is the transient response while the network is being lesioned. The third is the steady-state response after lesioning has ended. Colour coding is the same as in Figure 7. (c) The robot behaviour is displayed. We plot the swing height of the robot legs over time across the same three phases observed in the latent space. Lesioning is progressive and continues through the transient response period. Feet are abbreviated: LF (Left Front), RF (Right Front), LH (Left Hind), RH (Right Hind).

first phase, no lesioning has yet been applied and we observe normal dynamics. In the second phase, when the network is virtually lesioned, we see a transient response in the dynamics, where the latent trajectories become aberrant. We can represent the robot's behaviour by plotting the swing height of each foot (Figure S2c). Finally, the second phase (lesioning) dynamics persist into the third phase (steady-state response). In this phase we see that the Left Front and Right Hind legs lose their swing height. In other words, the robot begins to drag its legs on the ground as it tries to walk.

Although hidden units are zeroed in regular increments through the second phase of the

experiment, behaviour carries on for a while, until it fails rather suddenly. One hypothesis is that significant function is concentrated in specific hidden units: thus, zeroing these units causes shift in behaviour. Another hypothesis is that there is a capacity threshold: if the right number of neurons are damaged, then behaviour changes suddenly. But if we re-ran the experiment different 'critical neurons' would emerge.

To explore these hypotheses, we repeat the lesion experiment 200 times with different random seeds, the specific question being whether the critical change in behaviour corresponds across random seeds to the same number of hidden units being zeroed or not. We find that the number of hidden units that need to be zeroed before we observe the change in behaviour varies across the 200 times we repeated the experiment. In addition, once a critical neuron is zeroed, we find that normal function resumes if we restore that one unit by changing the mask from zero to one (i.e. 'rewinding' the damage). Together, these results suggest that damage to specific neurons accounts for the critical changes in behaviour.

If this is correct, what do these critical neurons do? To present one suggestive example, we observe that, for each repeat of the experiment, opposite front and back legs fail in behaviour together. The Left Front and Right Hind legs failed together more often (54.9%) than the Right Front and Left Hind legs (29.4%). In the remaining 15.7% cases, all four legs appear to fail at the same time. This suggest that the the lesioned neurons have learned shared representations for leg pairs. This makes sense given that the models are trained on trot gaits in which the movement of these legs are always correlated. If the models were trained with a wider variety of gaits, these correlations could be broken. In this case, we conjecture that lesioned neurons would show different patterns.
